# Screening of natural compounds that targets glutamate racemase of *Mycobacterium tuberculosis* reveals the anti-tubercular potential of flavonoids

**DOI:** 10.1038/s41598-020-57658-8

**Published:** 2020-01-22

**Authors:** Alka Pawar, Prakash Jha, Madhu Chopra, Uma Chaudhry, Daman Saluja

**Affiliations:** 1 Dr. B. R. Ambedkar Center for Biomedical Research, University of Delhi, Delhi, 110007 India; 2 Bhaskaracharya College of Applied Sciences, University of Delhi, Delhi, 110075 India

**Keywords:** Biochemistry, Computational biology and bioinformatics, Drug discovery, Drug discovery, Molecular biology

## Abstract

Tuberculosis (TB) is caused by *Mycobacterium tuberculosis* (MTB), a highly infectious disease accounting for nearly 1.5 million deaths every year and has been a major global concern. Moreover, resistance to anti-TB drugs is an arduous obstacle to effective prevention, TB care and management. Therefore, incessant attempts are being made to identify novel drug targets and newer anti-tubercular drugs to fight with this deadly pathogen. Increasing resistance, adverse effects and costly treatment by conventional therapeutic agents have been inclining the researchers to search for an alternative source of medicine. In this regard natural compounds have been exploited extensively for their therapeutic interventions targeting cellular machinery of MTB. Glutamate racemase (MurI) is an enzyme involved in peptidoglycan (PG) biosynthesis and has become an attractive target due to its moonlighting property. We screened various classes of natural compounds using computational approach for their binding to MTB-MurI. Shortlisted best docked compounds were evaluated for their functional, structural and anti-mycobacterial activity. The results showed that two flavonoids (naringenin and quercetin) exhibited best binding affinity with MTB-MurI and inhibited the racemization activity with induced structural perturbation. In addition, fluorescence and electron microscopy were employed to confirm the membrane and cell wall damages in mycobacterial cells on exposure to flavonoids. Together, these observations could provide impetus for further research in better understanding of anti-tubercular mechanisms of flavonoids and establishing them as lead molecules for TB treatment.

## Introduction

Tuberculosis is one of the pernicious diseases worldwide and has become a major global health concern due to ever evolving drug resistance. Even after about one hundred thirty years of discovery, *Mycobacterium tuberculosis* is one of the world’s major infectious killer^1^. More than 50,000 people every week die due to MTB infection and nearly one-third of the global population is asymptomatically infected^2^. Therefore, in 1993, the WHO promulgated TB as “a global health emergency”. However, even after a great strive to control TB globally, complete remission is still needed. Combinatorial therapy with anti-TB drugs has long been used as a promising strategy to effectively kill MTB, however, the emergence of drug resistance with drug tolerant strains has become the major hurdle^3^. Co-infection of TB with HIV^4^, as well as lack of patient compliance due to lengthy treatment protocol leads to challenges in diagnosis and treatment of TB^5^. Therefore, the requisite for new antibiotic targets as well as newer drugs are more exigent than ever before to combat antibiotic resistance menace.

The microbial cellular machinery mainly targets cell wall synthesis, gene expression and metabolic pathways. Among the limited options of targets, cell wall biosynthesis keeps the utmost widespread clinical utility efficacy for inhibitor designing. Targeting cell wall synthesis makes bacteria prone to rupture by osmotic pressure and therefore, inhibitors targeting cell wall biosynthesis proves to be bactericidal. The significant complex structure of the mycobacterial cell wall with the presence of virulence factors, makes MTB different from the other bacteria. The mycobacterial cell wall comprises of three layers which together form mycolyl-arabinogalactan-peptidoglycan (mAGP) complex. The innermost layer, Peptidoglycan (PG) layer is peculiar to bacterial kingdom and have been well-thought-out as an attractive target for drug designing. Based on the cellular localization of the enzymes, PG layer is synthesized in three distinct stages (I-III)^6^ and most of the drugs that have been clinically approved act by inhibiting the phase-III of cell wall biosynthesis. Isoniazid and Ethambutol are the only approved drugs which target cell wall biosynthesis by acting on mycolic acid and arabinogalactan layer respectively^7–9^. With increased bacterial resistance to compounds targeting phase III biosynthesis, phase-I pathway of PG biosynthesis is now contemplated as an alternate target for drug design^10^.

MurI gene encodes for Glutamate racemase enzyme involved in the initial stages of PG biosynthesis and therefore, becomes an attractive target for drug designing. Glutamate racemase involves in the inter-conversion of L- to D-glutamate (DGL), where DGL is a vital constituent of the PG layer formation^11,12^. In addition, Glutamate racemase (MurI) also has a profound role in sequestering DNA gyrase enzyme. Such proteins with two functions are called Moonlighting proteins. Moreover, accumulated evidence has shown that glutamate racemases are ubiquitous and are fairly conserved across bacterial kingdom. Furthermore, its absence in humans and other eukaryotes^13^ makes it an attractive target for drug discovery.

Since after the discovery of Rifampicin in the year of 1963, the pursuit for new anti- tubercular agents has been slow with the last two major drugs licenced several years ago *viz*., Bedaquiline^14^ and Delamanid^15^. Though both the drugs have lately acquired fast-tracked approval for use against multidrug resistance TB, these drugs have major side-effects. The emergent haul of antibiotic resistance, together with declined efforts in the expansion of new antibiotics impelled to the use of natural products as an alternative source for the development of anti-tuberculosis drugs, as they are exceptionally rich in chemical diversity with privileged antimicrobial activity^16^. Since ancient times, various parts of plants and their extracts have been used as medicines against many diseases. This traditional knowledge may be beneficial in designing future potent drugs^17^. Plants have plentiful metabolite mass and biosynthetic pathways which can be manipulated. With the availability of new tools and better understanding of genetics and biochemical pathways of microbes, natural products with curative action have proven to be priceless for the development of new drugs^18^. The bioactive natural compounds have been isolated from different plant species with a remarkable structural diversity, further inspiring development of new drugs for microbes. Amongst plethora of natural compounds, several flavonoids have shown to possess promising antimicrobial activities and can be explored to identify as antimycobacterial drug molecules. Flavonoids have shown to exert their effects by glutathione (GSH) enrichment, cytokine regulation^19^, activation of detoxification processes^20^, nucleic acid inhibitor and/or acting as a proteasome inhibitor^21^. However, the effect of flavonoids on the mycobacterial cell wall has not been well studied.

In lieu of their antimicrobial properties, we evaluated the effect of two shortlisted flavonoids on Mycobacteria and more specifically its enzyme MTB-MurI. The study provides evidence that both compounds inhibit the mycobacterial growth via hindering the cell wall biosynthesis. Based on these studies we propose that both naringenin and quercetin may turn out to be important leads for development of anti-TB drugs.

## Results

### Docking and molecular dynamics (MD) simulation

**Figure 1:**
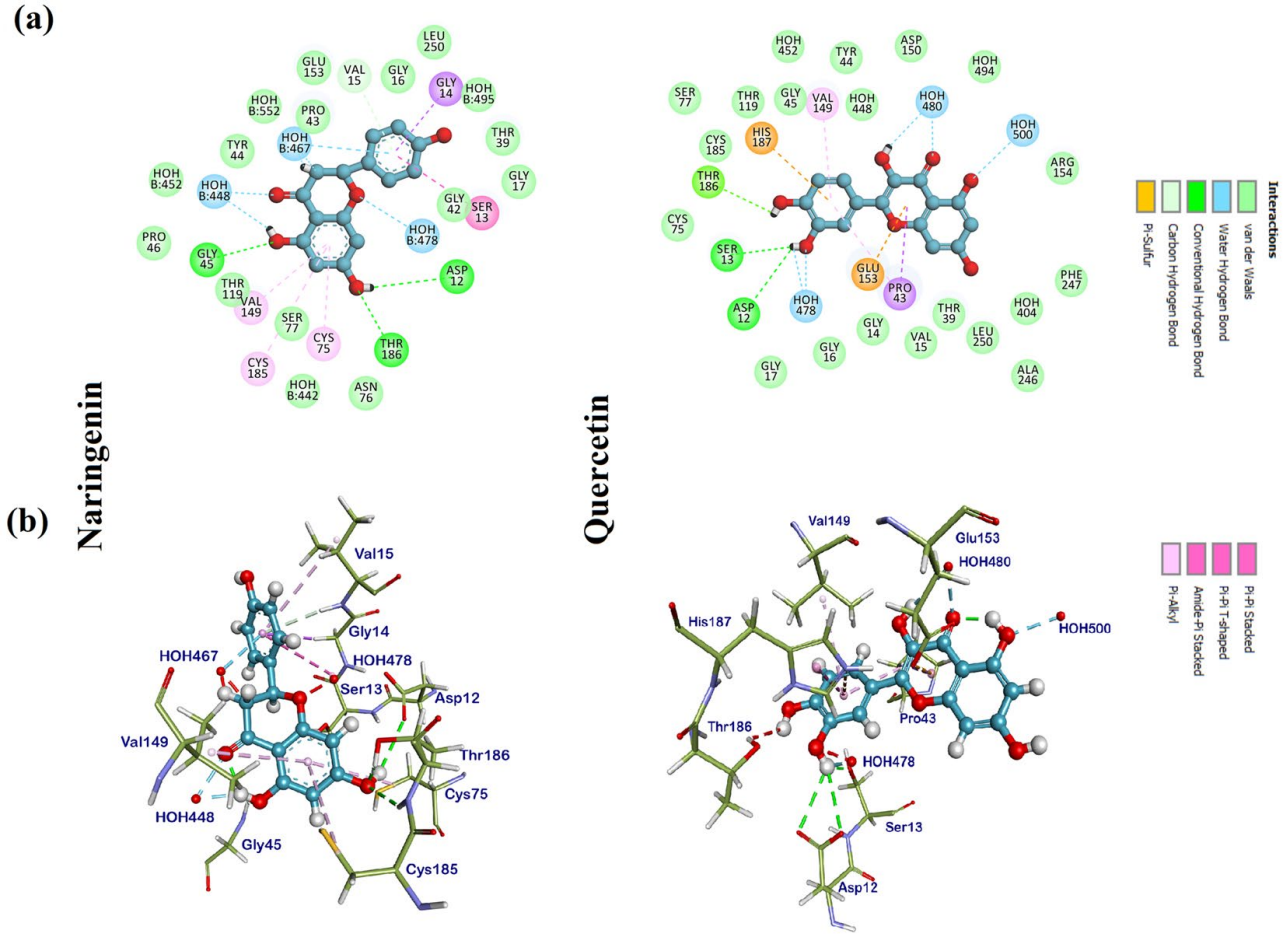
Molecular docking of flavonoid compounds onto the active binding site of MTB-MurI. (**a**) The 2D interaction diagram showing various types of interactions between MTB-MurI protein active site and (**b**) The 3D docking poses of naringenin and quercetin onto MTB-MurI protein. Key amino acid residues were determined in the 3D position in active sites.

Molecular docking is extensively used in drug designing by envisaging the experimental binding patterns as well as affinities of the lead compounds within the receptor active site. In the current study, we analyzed the molecular interactions of protein-ligand which was docked by Discovery studio (DS) 4.0, a commercial software followed by MD simulation studies. We have selected a wide range of various classes of natural compounds (Table S1)^22–31^ which were docked to identify potential compounds against MTB-MurI. Molecular docking studies were carried out to study the binding modes of natural compounds with MTB-MurI (PDB ID: 5HJ7)^32^. Out of the ten, three compounds namely, Curcumin, Rutin and Tannic acid did not dock into the receptor binding site. All the generated conformers from docking calculations were ranked in log file according to their CDOCKER (A CHARMm-Based MD Docking Algorithm) energy score. Docking results of the natural compounds along with control inhibitors i.e. D-glutamate, DGL and ethambutol, EMB (as described in our previous study, Pawar *et al*.)^33^ are summarized for the comparative purpose (Table S2). Based on the docking results, we shortlisted-two compounds, namely, naringenin and quercetin representing flavonoid class of compounds for further studies. Both naringenin and quercetin showed the interaction with Cys^75^ and Cys^185^, which are known to be involved in the catalytic mechanism of MTB-MurI and are expected to be responsible for the deprotonation of D and L-glutamate respectively^11,34^. In addition to these two interactions, both the compounds interacted with several other amino acid residues which are highly conserved among different bacterial species of glutamate racemases^32,35^. The relative stability of the compounds within the binding site was maintained due to the van der Waal’s interaction between the hydrophobic amino acids of the MTB-MurI protein and the ligands. The important interacting residues obtained during molecular docking of both naringenin and quercetin have been shown in Table S3, and their 2D and 3D interaction diagrams are shown in Fig. 1.

To evaluate the structural flexibility and stability of the protein-ligand interactions, the docked MTB-MurI complexes with both naringenin and quercetin were subjected to 10 ns MD simulations. The MD run resulted in removing steric clashes and optimization of molecular geometry of the complex. From the RMSD (Root mean square deviation) plots (Fig. S1a), we analysed that the deviation in the complexes was less than 2 Å (within the reliable range) throughout the simulation. Further, the RMSD of MTB-MurI with quercetin varies above 0.25 Å, while when complexed with naringenin, it converged at 0.2 Å. Greater RMSD of quercetin resulted in reorientation of quercetin within binding site while naringenin was intact to original pose (Fig. S2c). From the comparison of MD simulated poses with initial docked structures of flavonoids we observed that naringenin made several interactions with residues in MTB-MurI pocket (Ser^13^, Gly^14^, Val^15^, Asn^41^, Gly^42^, Pro^43^, Tyr^44^, Gly^45^, Ile^52^, Ser^77^, Ala^121^, Cys^185^, His^187^ and Val^199^) while quercetin interacted with Gly^14^, Val^15^, Asp^38^, Glu^153^, Arg^154^, Gly^155^, Ala^246^, Phe^247^ and Lys^249^. Among these, Tyr^44^, Cys^185^ and three catalytic water molecules which are important for inhibitor design, are always retained in naringenin but were altered in the case of quercetin (Table S2 and Fig. S2a,b). The average potential energy was calculated and found to be −7.15231e^5^ kJ/mol and −7.15007e^5^ kJ/mol for MTB-MurI-naringenin and MTB-MurI-quercetin respectively, depicting that both the complexes remain energetically stable throughout the simulation (Fig. S1b). Similar energies were observed with two control ligands used in our previous study *viz*., average potential energy of substrate D-glutamate complex MurI-DGL (−7.1718e^5^ kJ/mol) and inhibitor ethambutol complex, MurI-EMB (−7.1488e^5^ kJ/mol)^33^. The mobility of the flavonoids bound MTB-MurI protein was also analysed by calculating RMSF (Root mean square fluctuations) and was found to be in the range of 0–0.8 Å which shows negligible fluctuation (Fig. S1c).

However, the change in interactions between ligands and protein after MD run is due to several changes in binding pattern. The hydrogen bond distance between protein and ligands are enumerated in Table S2. For better clarity, we have also calculated the number of intermolecular hydrogen bonding (signifies a vital role in stabilizing the protein-ligand complexes) throughout the MD run for all the complexes i.e., MurI-DGL (positive control), MurI-ethambutol, MurI-naringenin and MurI-quercetin. The total number of H-bonds in all the complexes versus time at 300 K are shown in Fig. S3. MurI-DGL complex exhibited maximum twelve H-bonds during the simulation time period, which indicates that the D-glutamate is stable and having strong H-bond interactions with MurI receptor. Naringenin and quercetin are showing similar kind of intermolecular H-bonding pattern as ethambutol (reduction in the number of intermolecular H-bonding).

### Flavonoids inhibit the growth of Mycobacteria

**Figure 2:**
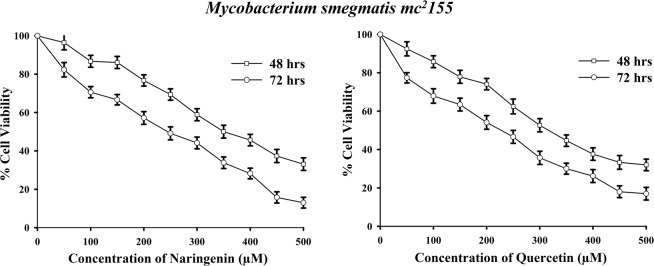
Effect of flavonoid compounds on viability of *Mycobacterium smegmatis* mc^2^155 cells. Effect of increasing concentrations of (**a**) naringenin and (**b**) quercetin was checked on cell viability of *M*. *smegmatis* cells after 48 hours and 72 hours using MTT assay to determine IC_50_ value. All values are expressed as mean ± SEM of three independent biological repeats.

To investigate the cell viability of mycobacteria in the presence of naringenin and quercetin, MTT assay was performed using *M*. *smegmatis* mc^2^155 cells. Interestingly, both naringenin and quercetin showed dose- and time- dependent decrease in cell viability of *M*. *smegmatis* cells (Fig. 2). The 50% growth inhibition (IC_50_) for naringenin was detected at 350 μM at 48 hours and 237.5 μM at 72 h, whereas, the IC_50_ for quercetin was found to be 312.5 μM at 48 hours and 225 μM at 72 h. These results clearly suggest that both the compounds showed cytotoxic effect on *M*. *smegmatis* mc^2^155 and inhibited its growth.

### Flavonoids induce cell death and membrane damage of Mycobacteria

**Figure 3:**
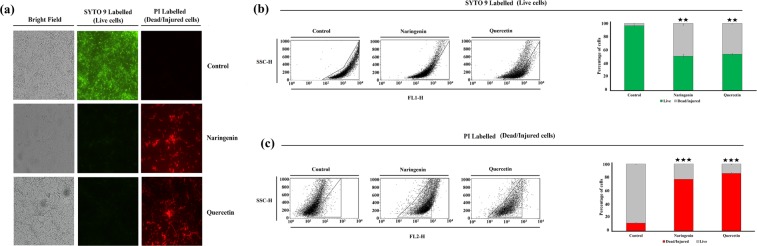
Flavonoids inhibit the growth and increase the cell death and membrane damage of *Mycobacterium smegmatis* mc^2^155. Cell death of *M*. *smegmatis* by naringenin and quercetin was determined using SYTO 9 and Propidium iodide (PI) by fluorescence microscopy and flow cytometry (**a**) Fluorescence microscopic images of representative naringenin and quercetin treated and untreated *M*. *smegmatis* cells. The cells were treated with naringenin and quercetin at their respective IC_50_ for 48 hours (350 µM/312.5 µM respectively). Bacteria in red are indicative of dead cells whereas, green indicates live bacteria. (**b**,**c**) A total of 10,000 cells were acquired for each flow cytometry analysis. Side scatter plot of *M*. *smegmatis* mc^2^155 cells labelled with either SYTO 9 or PI was collected for untreated and cells treated with naringenin and quercetin. Percentage of SYTO 9 and PI uptake is also shown by *M*. *smegmatis* cells as quantified from flow cytometry data. Results are presented as ± S.E.M of three independent experiments (*p ≤ 0.05; **p ≤ 0.02; ***p ≤ 0.001).

To delineate the mechanism behind the antimycobacterial activity of naringenin and quercetin, the ability of these compounds to permeabilize the bacterial membrane and inducing the cell death was examined using SYTO 9 and PI dyes which selectively stain live and dead cells, respectively. Cell death was investigated by both fluorescence microscopy as well as by flow cytometry. The representative fluorescence microscopic images show untreated and flavonoids treated cell population. The untreated *M*. *smegmatis* cells appeared predominantly bright green (demonstrating live cells); whereas both naringenin and quercetin treated cells substantially appeared red indicating dead cells (Fig. 3a). Similarly, the flavonoid-induced cell death of *M*. *smegmatis* cells were also determined using flow cytometry. In contrast to untreated cells, in cells treated with either naringenin or quercetin, a left shift of fluorescence intensity was observed in case of SYTO 9 staining whereas, an increased fluorescence intensity was observed in case of PI stain (Fig. 3b,c). Collectively, these results suggest significant cell death with naringenin and quercetin treatment (Fig. 3).

### Flavonoids exert no profound cytotoxicity in mammalian cells

Since, *Mycobacterium tuberculosis* mainly resides in the host macrophages, therefore, the effect of selected flavonoids on mammalian THP-1 (human monocytic macrophage) cells was investigated by MTT assay. As the results indicated, we found subtle cytotoxicity of the two flavonoids on these cells (Fig. S4).

### Flavonoids decrease the functional activity of MTB-MurI protein

**Figure 4:**
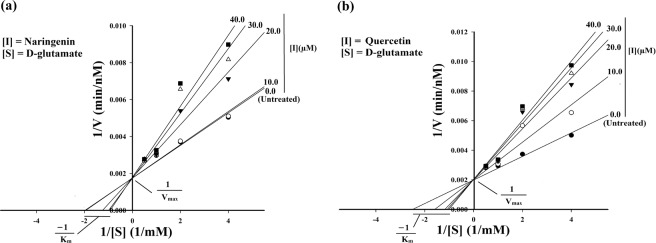
Lineweaver-Burk plot showing inhibitory effect of flavonoid compounds on the racemization activity of the purified MTB-MurI protein. Racemization activity of MTB-MurI was determined at different substrate concentrations in the absence and presence of various concentrations of (**a**) Naringenin and (**b**) Quercetin and Km and Vmax was calculated. Each data represents the value of three determinations (biological repeats).

**Figure 5:**
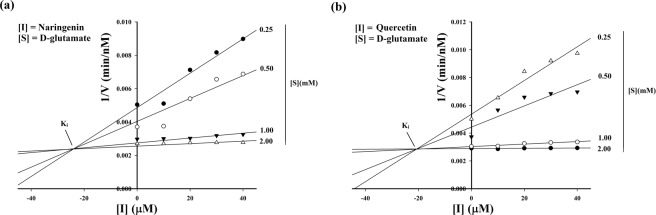
Dixon plot to determine the inhibitory effect of naringenin and quercetin on the purified MTB-MurI protein. Purified MTB-MurI was used for determining the Ki by Dixon plot at different substrate concentrations in the absence and presence of different concentrations of (**a**) Naringenin and (**b**) Quercetin. Each data represents the value of three determinations (biological repeats).

To explore the mechanism of action of both naringenin and quercetin, we studied the type of inhibition (competitive, uncompetitive, non-competitive or mixed) using the linear regression Lineweaver Burk plot to estimate the Michaelis constant, Km and maximum reaction velocity (maximum reaction rate), Vmax. The effect of naringenin and quercetin on enzyme activity was assessed through a coupled-enzyme assay by monitoring NADH at A_340_ for 6 minutes^11^. The recombinant purified MTB-MurI protein (35 µM), in the absence and presence of different concentrations (10–40 µM) of both naringenin and quercetin, was incubated with racemization buffer containing 10 mM D-glutamate and used for the formation of L-glutamate. The obtained kinetic parameters were plotted using the reciprocal of the reaction velocity (1/v on y axis) against the reciprocal substrate (1/[S] on x-axis) (D-glutamate) concentrations in the SigmaPlot Software 10.0. The value of Vmax of naringenin and quercetin was found to be 585.48 min^−1^ nm and 521.92 min^−1^ nm respectively. The Kcat values, largely unaffected in the presence of naringenin and quercetin, were found to be 0.1045 min^−1^ and 0.0932 min^−1^ respectively (Fig. 4a,b). The calculated Kinetic parameter, Km showed an increase in the presence of the inhibitors (Table 1). The inhibition constant, Ki, for both the compounds was also calculated which gives an indication of how potent an inhibitor is. The Ki of naringenin was found to be 23.814 µM whereas, of quercetin was 20.8082 µM (Fig. 5a,b).

**Table 1:** Michaelis constant (Km) - an enzyme activity parameter of MTB-MurI.

Concentration (mM)	Naringenin	Quercetin
**Michaelis constant**, **Km (mM)**		
None	0.49895	0.4117
10	0.5078	0.6330
20	0.8160	0.8480
30	0.9924	0.9391
40	1.0645	0.9817

Functional activity parameter, Michaelis constant (Km) of the purified MTB-MurI protein in the absence and presence of varying concentrations of naringenin and quercetin.

### Flavonoids induce structural change in MTB-MurI protein

**Figure 6:**
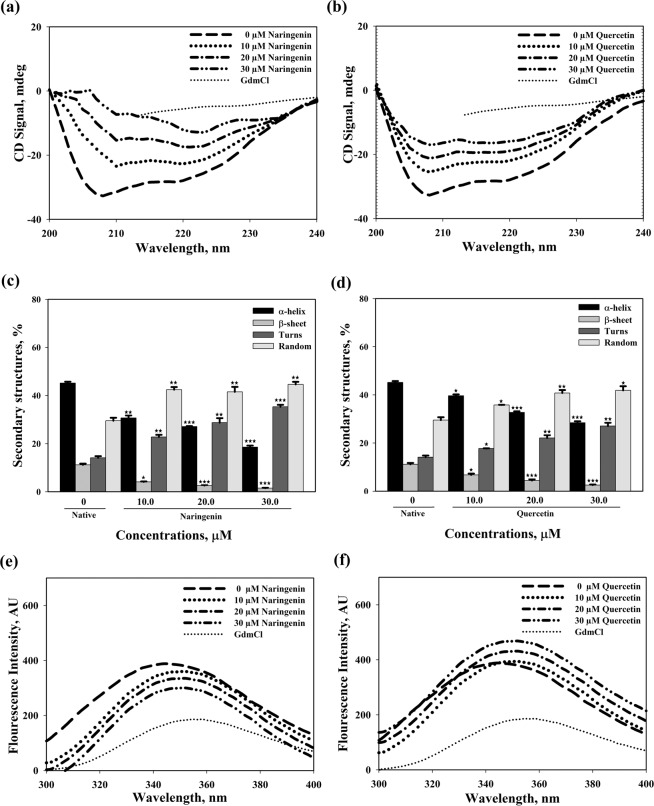
Effect of flavonoid compounds on the structure of purified MTB-MurI protein. (**a**,**b**) Far-UV CD spectra, (**c**,**d**) percent secondary structures of native protein alone and in the presence of naringenin and quercetin (**e**,**f**) intrinsic fluorescence spectra of the purified MTB-MurI protein in the absence and presence of different concentrations of naringenin and quercetin. Excitation wavelength of 268 nm was used for the protein and emission was recorded at the wavelength range of 290–450 nm. Purified protein with DGL (D-glutamate) was used as control. Data represented after normalizing with that of native protein. Results are presented as ± S.E.M of three independent experiments (*p ≤ 0.05; **p ≤ 0.02; ***p ≤ 0.001).

To assess the effect of flavonoid compounds on native state structural integrity of MTB-MurI protein, CD spectroscopic studies were employed. The effect of varying concentrations of both naringenin and quercetin on the secondary structure of MTB-MurI was determined with Far-UV CD spectra. Secondary structure of the untreated MTB-MurI protein displayed negative peaks at 208 and 222 nm, the characteristic peaks representing α-helices. However, in the presence of naringenin or quercetin, a concentration dependent decrease in secondary structure of MTB-MurI was observed (Fig. 6a,b). In the presence of 30 μM of naringenin a dramatic loss of both 208 and 222 nm peaks was observed, suggesting complete loss of secondary structure of MTB-MurI. However, in the presence of 30 μM of quercetin, MTB-MurI protein retained some structure. We also analyzed Far-UV spectra to quantitate the percent loss of secondary structure in terms of α-helix using in-built software of the CD-spectrophotometer. Our results indicated that the secondary structure of untreated MTB-MurI contained 45.16 ± 0.8% of α-helix. Naringenin and quercetin perturbed the secondary structure of MTB-MurI by reducing the helical contents by 73.87% (from 45.16% to 11.8 ± 0.5%) and 58.9% (from 45.16% to 18.56 ± 0.9%) respectively (Fig. 6c,d). This is further substantiated by another result where flavonoids are found to have a subvert consequences on the tertiary structure of the MTB-MurI protein with regard to the environment of tyrosine residues (Fig. 6e,f). There is an explicit spectral shift in λmax (345 nm) of MTB-MurI protein towards higher wavelength (360 nm, bathochromic shift) along with alterations in the fluorescence intensity with increasing concentrations of both naringenin and quercetin.

### Flavonoid treatment induces morphological changes and cell wall damage of Mycobacteria

**Figure 7:**
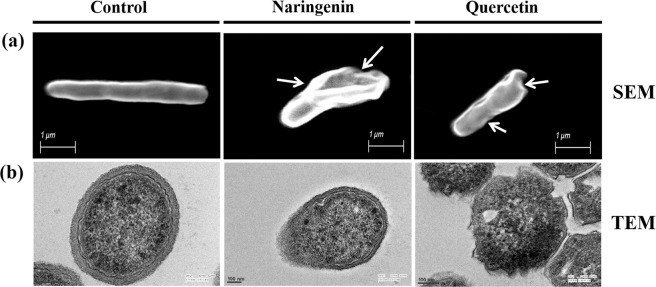
Scanning and transmission electron micrographs of *M*. *smegmatis* mc^2^155 showing cell damage in the presence of naringenin and quercetin. The cells were treated with naringenin and quercetin at their respective IC_50_ for 48 hours (350 µM/312.5 µM respectively). (**a**) Representative images of mycobacterial cells in the absence and presence of quercetin and naringenin by SEM showing alterations in cell morphology and outer layer. (**b**) Representative TEM images of *M*. *smegmatis* cells showing eroded cell surface in the presence of quercetin and naringenin as compared to that of the control cells. Bar: 1 µm for SEM analysis and 100 nm for TEM.

Since the flavonoids showed inhibitory effect on racemization activity of MTB-MurI protein, we were further avid to check the effect of these flavonoids on the alterations in cell morphology using electron microscopy. Scanning electron microscopy (SEM) of flavonoid treated cells of *M*. *smegmatis* mc^2^155 cells clearly revealed distorted morphology with depressions on the surface. The cell surface also had irregular/wrinkled appearance. However, the untreated cells had smooth, undamaged cell wall and were devoid of any distinct morphological alterations (Fig. 7a).

Furthermore, to analyze the effect of flavonoids on the cell wall integrity, *M*. *smegmatis* cells were examined by transmission electron microscopy (TEM). A smooth, gentle and consistent cell surface revealed a multi-layered (teichoic) organization of the mycobacterial cell wall (Fig. 7b). However, naringenin and quercetin treated cells exhibited a diffused, scattered, eroded, uneven and frayed outer layer, likely indicating the loss of peptidoglycan layer (Fig. 7b).

## Discussion

Multidrug-resistant MTB has resulted in considerably high mortality rates with restricted or no alternatives for the treatment^36^. Hence, novel and promising drugs and drug targets are of absolute requisite for tuberculosis with a possibility of increased clinical efficiency. Furthermore, the adverse effects of some of the drugs and the cost of currently available treatment has increased the inclination towards the use of an alternative source of medicine by exploring natural compounds as they are less or non-toxic to the host system. Target based screening of small molecules is an important tool in drug discovery pipeline. Most of the time, in the pursuit for developing new inhibitors for any target protein, *in silico*-based screening of compounds followed by docking on the active binding site is used. These compounds are then shortlisted based on the final scores and ranked according to their stability and affinity for the protein. The potential leads are further tested *in vitro* and *in vivo* before they can be recommended for clinical trials. Due to ever evolving drug resistance problem, research is being carried out worldwide to focus upon the identification of anti-TB drugs and novel drug targets. One promising target for tuberculosis treatment is MTB-MurI. Beside the essential role of Glutamate racemase (MurI) in peptidoglycan (PG) biosynthesis, evidences have also shown its profound role in sequestering DNA gyrase enzyme. Such proteins with two functions are called Moonlighting proteins. Both cell wall biosynthesis and DNA gyrase are the essential functions, therefore, one can assume that if any drug targets two pathways simultaneously, it will be more effective in inhibiting the bacterial growth. Moreover, accumulated evidences have shown that glutamate racemases are ubiquitous and are fairly conserved across bacterial kingdom. Furthermore, its absence in humans as well as in other eukaryotes makes it an ideal target for drug discovery.

In recent past, MurI in *Staphylococcus aureus* and *Helicobacter pylori* was shown to be inhibited by 8-benzyl pteridine-6,7-diones and pyridodiazepine amines respectively^37,38^. Inhibitory effect of some of the benzoxazole and indazole derivatives were also reported against TB^39,40^. However, extensive studies are not in place to establish these compounds as inhibitors of racemization activity of MTB-MurI. In the current study, using systematic approach, we have identified flavonoids as anti-tubercular compounds followed by elucidation of their mechanism of action.

**Figure 8:**
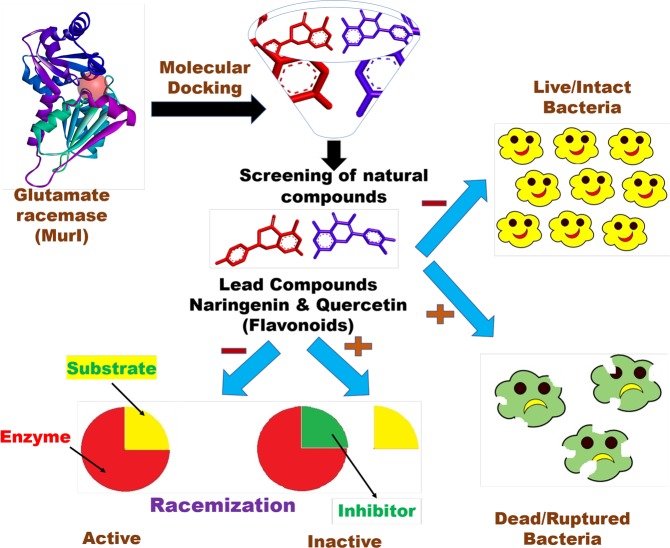
Flavonoid-induced damage of mycobacterial cell wall through structural and functional inhibition of MTB-MurI. A pictorial image showing brief, pictorial and visual summary of the main findings of the study.

It was noticed that seven out of ten compounds docked successfully with MTB-MurI. Naringenin and quercetin ranked higher as compared to rest of the compounds as the most potent inhibitors. Using computational and CD-spectrophotometric approach we demonstrated that the binding of naringenin and quercetin to MTB-MurI significantly influenced the conformation of the protein. *In silico* docking studies revealed that both naringenin and quercetin interact with important residues suggesting that these compounds bind strongly at the substrate binding site. The interacting residues involve, Asp^12^, Cys^75^, Cys^185^ and His^187^, where the two cysteine residues are involved in the acid/base catalysis during the inter-conversion of glutamate enantiomers^12,34^. To evaluate the flexibility and the binding affinity of the inhibitors with the MTB-MurI protein, a 10 ns molecular dynamics (MD) simulation of the docked complexes was carried out. We observed that the binding patterns obtained after MD simulation were energetically stable throughout the simulation. However, conformational changes of the ligands in both the complexes were related to the side chains rotations and this observation can be correlated with our earlier studies as both naringenin and quercetin also exhibited similar kind of mechanism like ethambutol^33^. These inhibitors may first bind to the active site of the MTB-MurI and subsequently change its conformation as also evidenced by our CD and fluorescence studies. Such kind of repositioning of ligand causes rearrangement of binding pocket of MTB-MurI. The CD-spectroscopic studies using purified protein clearly indicated that binding of naringenin and quercetin triggered a structural change in MTB-MurI protein, both at secondary and tertiary levels. Structural alterations in MTB-MurI were also observed using fluorescence spectrophotometry wherein a bathochromic shift in the fluorescence emission profile was observed along with change in the intensity (Fig. 8). It is pertinent to mention here that MurI lacks tryptophan residues and although we excited the protein at 280 nm wavelength, the emission maxima was found at 345 nm and not at 310 nm (typical for tryptophan). Previous reports have suggested that proteins with multiple tyrosine lacking tryptophan residues, display emission peak at ~345 nm because of the generation of tyrosinates^33,41^.

Once established that naringenin and quercetin can bind to MTB-MurI and can serve as potential inhibitors of Mycobacterial growth, we examined the effect of these compounds on the enzymatic action of mycobacterial MurI using purified protein. A racemization activity was performed and enzymatic kinetic parameters (Km and kcat) of the protein in the presence and absence of naringenin and quercetin were calculated. Both the compounds inhibited the racemization of D- to L-glutamate in a concentration dependent aspect. A notable increase was observed in Km without much change in Kcat. Detailed kinetic studies indicated an inhibition constant Ki at 23.814 µM, 20.8082 µM for naringenin and quercetin respectively. We envisage that both naringenin and quercetin competitively inhibit MurI enzyme (Fig. 8). The racemization function and enzymatic constants of MTB-MurI was in the range as reported for other bacteria. Although, the Michaelis-Menten constant of MTB-MurI was almost equivalent to that of *A*. *pyrophilus*^42^ and *L*. *fermenti*^11^, the Catalytic constant was lower as compared to these bacterial species.

Viability estimation is of critical importance in evaluating antimicrobial activity and their efficiency. In the present study a relatively fast growing and non-pathogenic strain, *M*. *smegmatis* was used. More than 50% of genes (2000 genes) are homologous among MTB and *M*. *smegmatis*. Importantly, *M*. *smegmatis* also shares peculiar cell wall characteristic of MTB. The cell wall of both MTB and *M*. *smegmatis* is composed of three layers containing mAGP complex. The innermost layer i.e. peptidoglycan (PG) layer is conserved throughout the bacterial kingdom. In both MTB and *M*. *smegmatis*, the lowest layer of the mAGP complex consists of alternating N-acetylglucosamine (GlcNAc) with modified muramic acid (Mur) residues, linked in a β (1 → 4) configuration. The overall cell wall assembly in both MTB and *M*. *smegmatis* is identical. Hence, we used the non-pathogenic strain, *M*. *smegmatis* as the model organism to check the effect of both naringenin and quercetin onto the cell wall. Additionally, the MurI enzyme used in the current study is conserved in both the organism. The MurI of *M*. *smegmatis* shares 87.80% identity and 94.1% similarity with MTB (calculated using BLASTp, NCBI) at the protein level) and the binding site is completely conserved. Therefore, viability assays were performed using *M*. *smegmatis*. Our results showed that these flavonoids induced membrane permeabilization and cell wall damage. We observed dose dependant killing of mycobacterial cells with an IC_50_ concentration of 350 μM and 312.5 μM at 48 hours of naringenin and quercetin respectively. We also provide evidence for the loss of integrity of the mycobacterial membrane by the flavonoids using different methods; (1) fluorescence microscopy studies using SYTO 9 and PI probes showed excessive entry of PI molecules in mycobacterial cells (2) flow cytometry indicated disruption of the membrane as we observed an increased PI uptake in naringenin, and quercetin treated cells. (3) Electron microscopy results clearly showed membrane disruption and cell wall damage of the mycobacteria. Taken together, our results suggested a distinct relationship among mycobacterial killing along with its membrane damage due to flavonoid compounds.

## Conclusion

By using computational approaches and validating the observations by subsequent *in vitro* experiments, we have identified two potential molecules against MTB-MurI. These lead compounds can provide a platform for further modifications and detailed efficacy analysis leading to discovery/designing of new drugs against antibiotic resistant mycobacterium. Our study provides an impetus for future research in discovering novel flavonoids as antimicrobial agents against resistant microbes.

## Experimental Methods

### Docking and molecular dynamics (MD) simulation

The docking studies were performed using CDOCKER tool of Discovery Studio (DS) 4.0. The crystal structure of MTB-MurI with bound D-glutamate (DGL) (PDB ID: 5HJ7) was selected as a receptor and catalytic site for docking was determined based on co-crystallised ligand i.e., DGL^32^. Several natural compounds were initially selected from different class and their.sdf files were obtained from PubChem on NCBI database. Both receptor (MTB-MurI) as well as ligands were prepared before docking in DS 4.0. A total 10 docked conformers were generated for each ligand in docking results and these conformers were arranged according to their binding energies. To get the actual binding modes, the selected docked complexes with lowest docking energy were subjected to MD simulation with Gromacs 4.5.6. The GROMOS96 forcefield and PRODRG server was applied to describe the protein and ligand respectively. The model was immersed in water box of cubic shape and the complexes were solvated and minimized using steepest descent algorithm. The energy minimized complexes were equilibrated under NVT and NPT (constant Number of particles, Volume, and Temperature) condition to 100 ps. Finally, a 10 ns of MD run was performed with time step of 2 fs. Post MD simulation analysis were carried out in PyMOL and Xmgrace software.

### MTT based whole cell assay for IC_50_ determination

IC_50_ determination was carried out using *M*. *smegmatis* strain mc^2^155 cells grown in 7H9 media (Difco, Middlebrook from Becton Dickinson and Company, Sparks, MD, USA) having 0.2% glycerol and 0.05% Tween 80 and supplemented with 10% OADC^43^. Appropriate dilutions (0 to 500 µM) of both naringenin (N5893) and quercetin (Q4951); Sigma-Aldrich, St Louis, MO, USA, were added to the 7H9 media containing *M*. *smegmatis* cells and incubated for 48 hours and 72 hours in an assay volume of 200 μl and IC_50_ values were calculated by using MTT assay (M6494; Invitrogen, Life Technologies). Ethambutol was used as a positive control. The values were calculated from at least three independent experiments and represented as Mean ± S.E.M. Obtained IC_50_ concentration at 48 hours was used for all the subsequent experiments.

### Microscopic and FACS evaluation of mycobacterial viability

Bacterial viability and membrane integrity were analyzed using SYTO 9 dye (S34854; for live cells) and Propidium Iodide (PI, P3566; for dead cells) from Invitrogen, for microscopy and FACS analysis. The bacterial cells treated with either naringenin or quercetin for 48 hours were co-stained with SYTO 9 and PI. Live and dead cells were observed at obtained IC_50_ under the fluorescence microscope according to the protocol suggested by the manufacturer. The samples were incubated for 15 minutes in dark at room temperature and 10 μl of this sample was used for the microscopy. The slide was viewed under a fluorescence microscope (Motorized Inverted Microscope. Ii2; Nikon), using 60X objective sequentially using fluorescence setting for FITC (green/SYTO 9) and PI (red/PI) filters, respectively, followed by phase contrast and bright field settings.

For flow cytometry analysis, the bacterial cells treated with either naringenin or quercetin for 48 hours were stained separately with SYTO 9 and PI. Scatter characteristics of cells were analysed by flow cytometry analysis (FACS Calibur, Becton Dickinson) using unfixed cell suspension. The two stains SYTO 9 and PI were used individually to capture the SSC plot in the study and the instrument was set to detect 10,000 cells (events) per sample. Side scatter (SSC) were detected using linear amplification. Fluorescence detection used logarithmic amplification (FL1 (green) = 530/30 nm; FL2 (red) = 585/40 nm). Untreated cells, where no drug treatment was given, served as control.

### Cytotoxicity determination

Toxicity profile of both the compounds was determined with inhibition assay using human monocytic macrophage cell line (THP-1 cells; a kind gift from Prof. K. Natarajan, ACBR, University of Delhi, India). Cells were incubated for 48 hours at 37 °C in medium containing varying concentrations of flavonoid compounds in sterile 96-well microtitre plates. The growth media used was RPMI 1640 (R0883; Sigma-Aldrich, St Louis, MO, USA) containing 10% fetal bovine serum (10500064; Gibco, Carlsbad, CA, USA), Antibiotic-Penstrep (15070063; Invitrogen, Life Technologies) and 2 mM L-glutamine (G6392; Sigma-Aldrich, St Louis, MO, USA). Three independent experiments were carried out for determining IC_50_ using MTT reagent and values are represented as Mean ± S.E.M.

### Effect of flavonoids on the racemization activity of MTB-MurI

The ability of the flavonoids to inhibit the functional (racemization) activity of MTB-MurI was determined as described earlier^11^. MTB-MurI was over expressed in *E*.*coli* using plasmid pQE-30 Xa and expression vector M15 [pREP4] with N-terminal histidine tags and purified from the inclusion bodies using N-Lauroylsarcosine sodium salt. The purity and the molecular mass of the purified recombinant protein was confirmed using SDS-PAGE and western blot analysis respectively as previously shown^33^. Purified MTB-MurI protein (35 µM) in the absence and presence of different concentrations (10–40 μM) of naringenin or quercetin was tested for the formation of L-glutamate as described earlier^11^. The product formation was then checked by adding 5 mM NAD^+^ (N3014; Sigma-Aldrich) and 10 U of GDH (G2626; Sigma-Aldrich) followed by measuring absorbance at 340 nm for 6 minutes using Jasco V-660 UV/Vis spectrophotometer equipped with a Peltier type temperature controller.

For determining the effect of compounds on the kinetic parameters (Km, Michaelis constant and kcat, catalytic constant), racemization activity was also measured using D-glutamate as a substrate. For this, purified MTB-MurI protein (35 µM) was incubated with different concentrations of substrate (0.25–2.5 mM) and with or without the flavonoid compounds (10–40 µM). It is pertinent to mention here that enzymatic reactions in the absence or presence of flavonoid compounds were observed to be completed in 6 minutes. The initial velocity (v) was deduced from the slope of the linear part of the recordings, usually first 30 s and was used for measuring specific activity. The reciprocal plot of reaction velocity (1/v at y axis) against the reciprocal D-glutamate, substrate concentrations (1/[S] at x-axis) was analyzed for Km and Vmax using the relation (Eq. 1). From this analysis the values of kcat were also determined.

1 $${\rm{v}}=\text{Vmax}[S]/({\rm{Km}}+[{\rm{S}}])$$

The inhibitor constant, Ki, for both the compounds was also calculated using the Lineweaver-Burk plot. All the measurements were recorded in triplicate and the Mean ± S.E.M was calculated from the curve using SigmaPlot Software 10.0.

### Effect of flavonoids on the structural changes of MTB-MurI

For determining the secondary structural alterations of MTB-MurI in the presence of flavonoid compounds, CD measurements were recorded for Far- UV CD spectra in a Jasco J-810 spectropolarimeter equipped with a Peltier-type temperature controller. Purified MTB-MurI protein (0.5–0.6 mg/ml) was incubated overnight with different concentrations of either quercetin or naringenin. Secondary structure estimation from the Far-UV CD spectra was calculated using Yang’s method^44^. Additionally, for determining changes in tertiary structure in terms of aromatic amino acid, fluorescence spectra of the purified MTB-MurI protein (0.1 mg/ml) in the absence and presence of either naringenin or quercetin was measured. Perkin Elmer LS 55 Spectro-fluorimeter with both excitation and emission slits set at 10 nm in a 3 mm quartz cuvette was used. For tyrosine fluorescence measurements, MTB-MurI protein was excited at 268 nm, while the emission spectra were recorded from 290 to 450 nm. All the measurements were recorded in triplicate with subtraction of necessary blanks.

### Effect of flavonoids on the cell wall damage

To check the effects of flavonoids on the bacterial cell wall damage, electron microscopy analysis was performed. *M*. *smegmatis* mc^2^155 culture was grown for 48 hours in 7H9 broth in the absence and presence of naringenin and quercetin at a concentration of 350 μM and 312.5 μM respectively. For scanning electron microscopy (SEM), the cells were fixed overnight at 4 °C in a mixture of 2% paraformaldehyde and 2.5% glutaraldehyde in 0.1 M phosphate buffer (pH 7.4). A drop of sample was spreaded on a cover slip, air-dried, sputter-coated with colloidal gold and the morphology was examined under scanning electron microscope (EVO18 SEM, Zeiss) at an operating voltage of 15 kV^45,46^.

To carry out transmission electron microscopy (TEM), flavonoid-treated *M*. *smegmatis* cells were embedded in araldite CY 212 (TAAB kit, UK) and cut into ultrathin sections. The sections were stained with aqueous toluidine blue and observed under a light microscope for gross observation of the area and quality of the tissue fixation. Thin sections of grey-silver colored interference (70–80 nm) were cut and mounted onto 300 mesh-copper grids. Sections were stained with alcoholic uranyl acetate and alkaline lead citrate for 10 minutes in each, washed gently with distilled water and observed under a Tecnai G2 20 high resolution transmission electron microscope (Fei Company, The Netherlands) at an operating voltage 200 kV^47^. Images were digitally acquired by a CCD camera (Megaview III, Fei Company) attached to the microscope using TIA software.

## Supplementary information

Supplementary Information
