# Supplementary material for: Screening of natural compounds that targets glutamate racemase of Mycobacterium tuberculosis reveals the anti-tubercular potential of flavonoids
Source: Sci Rep. 2020 Jan 22;10:949. doi: 10.1038/s41598-020-57658-8 (PMC6976638; doi:10.1038/s41598-020-57658-8)
Supplement: Supplementary file 1 — Supplementary Information [file 41598_2020_57658_MOESM1_ESM.pdf]

Supplementary Information

Screening of natural compounds that targets glutamate racemase of *Mycobacterium tuberculosis* reveals the anti-tubercular potential of flavonoids

Alka Pawar[1, 2], Prakash Jha[1], Madhu Chopra[1], Uma Chaudhry [2], Daman Saluja[1*]

[1]Dr. B. R. Ambedkar Center for Biomedical Research, University of Delhi, Delhi-110007, India
[2]Bhaskaracharya College of Applied Sciences, University of Delhi, Delhi-110075, India

Correspondence: Daman Saluja

Email Address: (dsalujach59@gmail.com),

Full Postal Address: Dr. B. R. Ambedkar Center for Biomedical Research, University of Delhi, Delhi-110007, India.

Phone No.: 91-11-27666272, 27667151

Name of the Natural Compound used in the study	Classes	Targeting bacterial pathogens	References
Quercetin, Naringenin & Rutin	Flavonoids	• Inhibition of cell wall envelope of *Helicobacter pylori* and *Mycobacterium tuberculosis*.<br>• Inhibition of nucleic acid synthesis of *E. coli*, *Mycobacterium tuberculosis* and *Mycobacterium smegmatis*	[22]
Coumarin, Esculetin & Umbelliferone	Coumarin derivatives	• Inhibition of quorum sensing and biofilm formation against *Staphylococcus aureus*, *Salmonella enterica*, *Enterobacter cloacae and Helicobacter pylori*	[23]
Gallic acid	Tri-hydroxy phenolic acid	• Inhibition of bacterial cell wall of *Staphylococcus aureus*, *E. coli*, and *Pseudomonas aeruginosa*	[24]
Tannic acid	Tannin	• Inhibition of biofilm formation and plasma coagulation against *Staphylococcus aureus*<br>• Inhibition of adhesion and biofilm formation against *Pseudomonas aeruginosa*	[25-27]
Caffeic acid	Hydroxy cinnamic acid	• Inhibition of membrane permeabilization, intracellular potassium ion efflux, and nucleotide leakage against *Pseudomonas aeruginosa*<br>• Inhibition of the β-class carbonic anhydrases with carboxylic acids from *Mycobacterium tuberculosis*	[28-29]
Curcumin	polyphenol	• Acts as a potent inducer of apoptosis-an effector mechanism used by macrophages to kill intracellular *Mycobacterium tuberculosis*<br>• Damaging of bacterial membrane against *E. coli* and *Staphylococcus*	[30-31]

Table S1: Classification of various class of natural compounds along with their antimicrobial activity.

Compounds	Docking Scores										
	(-) CDOCKER Energy (kcalmol^{-1})	(-) CDOCKER interaction Energy (kcalmol^{-1})	Lig_1	Lig_2	-PLP1	-PLP2	Jain	-PMF	-PMF04	LUDI 1	LUDI 2	LUDI 3
Quercetin	26.927	30.499	4.28	4.79	70.73	71.46	0.54	82.28	31.75	298	258	345
Naringenin	18.848	28.048	3.13	4.36	59.31	62.71	3.2	84.39	30.73	383	357	432
Esculetin	12.425	15.357	2.71	3.51	48.22	47.99	2.2	70.32	19.55	327	324	409
Gallic acid	10.965	16.659	4.45	4.14	52.14	64.05	3.33	64.67	26.33	364	306	342
Umbelliferone	12.492	18.127	2.45	3.47	42.71	47.14	3.12	58.07	8.32	352	320	409
Caffeic acid	16.093	16.24	-999.9	-999.9	35.87	36.06	-0.57	27.71	13.12	196	217	190
Coumarin	13.891	18.227	2.17	3.47	46.55	45.43	2.71	54.61	9.77	364	314	401
Curcumin	Not docked onto the MTB-MurI											
Rutin												
Tannic acid												

Table S2: Molecular docking scores of various natural compounds against MTB-MurI. Screening of various classes of natural compounds using molecular docking analysis against MTB-MurI. DGL and EMB used as controls. **CHARMm forcefield-based scoring functions= CDOCKER Scores**; **Empirical fitting approach** = Jain, Ligscore, and Ludi; **Knowledge based statistical approach** = Potential of Mean Force (PMF), PMF04; **Correlation with binding affinity** = Piecewise Linear Potential (PLP)

Type of Interactions	Binding interactions post docking (pre MD simulation)	Binding interactions post MD-simulation
**Naringenin**		
**Hydrogen Bond**	Asp^{12} (2.88 Å), Val^{15} (3.00 Å), Gly^{45} (2.13 Å) and Thr^{186} (2.15 Å)	Ser^{13} (2.59 Å), **Asn^{41}** (2.70 Å), Pro^{43} (2.83 Å), Tyr^{44} (1.61 Å) and Ser^{77} (2.96 Å).
	HOH^{448}, HOH^{476} and HOH^{478}	HOH^{448} and HOH^{467}
**Van der Waals**	Gly^{16}, Gly^{17}, Thr^{39}, Gly^{42}, Pro^{43}, Tyr^{44}, Pro^{46}, Asn^{76}, Ser^{77}, Thr^{119}, Glu^{153} and Leu^{250}	Val^{15}, Gly^{42} and Gly^{45}
	HOH^{442}, HOH^{452}, HOH^{495} and HOH^{552}	HOH^{478}
**Hydrophobic**	Ser^{13}, Gly^{14}, Val^{15}, Cys^{75}, Val^{149} and Cys^{185}	Gly^{14}, Tyr^{44}, **Ile^{52}**, **Ala^{121}**, Cys^{185}, His^{187} and Val^{l99}
**Quercetin**		
**Hydrogen Bond**	Asp^{12} (2.22 Å), Ser^{13} (2.54 Å) and Thr^{186} (2.15 Å)	Asp^{28} (2.50 Å)
	HOH^{478}, HOH^{480} and HOH^{500}	HOH^{448}, HOH^{467} and HOH^{480}
**Van der Waals**	Gly^{14}, Val^{15}, Gly^{16}, Gly^{17}, Thr^{39}, Tyr^{44}, Gly^{45}, Cys^{75}, Ser^{77}, Thr^{119}, Asp^{150}, Cys^{185}, Ala^{246}, Phe^{247} and Leu^{250}	Gly^{14}, Val^{15}, Glu^{153}, **Gly^{155}** and **Lys^{249}**
	HOH^{404}, HOH^{452} and HOH^{494}	
**Hydrophobic**	Pro^{43}, Vall^{49}, Glu^{153} and His^{187}	**Arg^{154} and Ala^{246}**

Table S3: Binding interacting residues of MTB-MurI in the presence of flavonoid compounds. Various key binding residue interactions of MTB-MurI protein with quercetin and naringenin during molecular docking analysis and after molecular dynamics simulation. The residues highlighted in bold are the newly added residues after MD simulation.

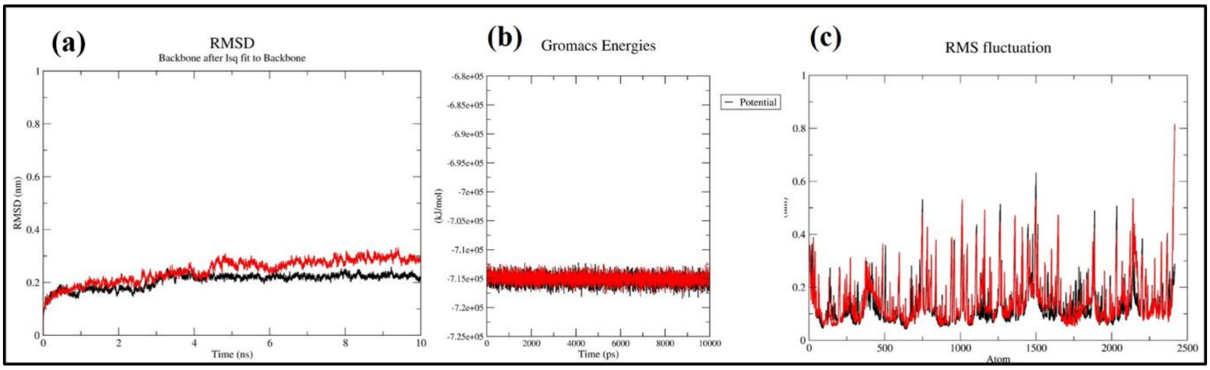

Fig. S1: Molecular Dynamics Simulation of flavonoids onto the active binding site of MTB-MurI: (a) Backbone RMSDs for both quercetin and naringenin docked onto MTB-MurI structure at 300K **(b)** Potential energy (kJ/mol) of quercetin and naringenin docked onto MTB-MurI structure **(c)** RMSF of the backbone atoms of both naringenin and quercetin docked onto glutamate racemase structure vs. time at 300K. Red colour indicated naringenin and Black colour represents quercetin.

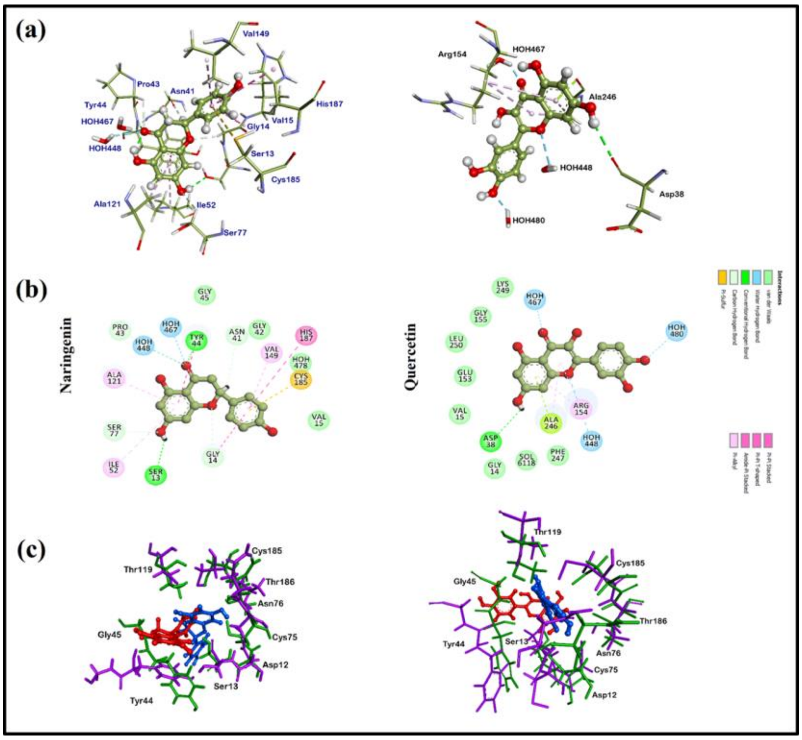

Fig. S2: Molecular Dynamics Simulation of flavonoids onto the active binding site of MTB-MurI: Molecular docking of naringenin and quercetin onto the active binding site of MTB-MurI after MD simulation. **(a)** The 3D and, **(b)** The 2D interaction diagram showing interactions between MTB-MurI active site naringenin and quercetin and **(c)** superimposition of both the ligands before and after MD simulation with catalytically important residues.

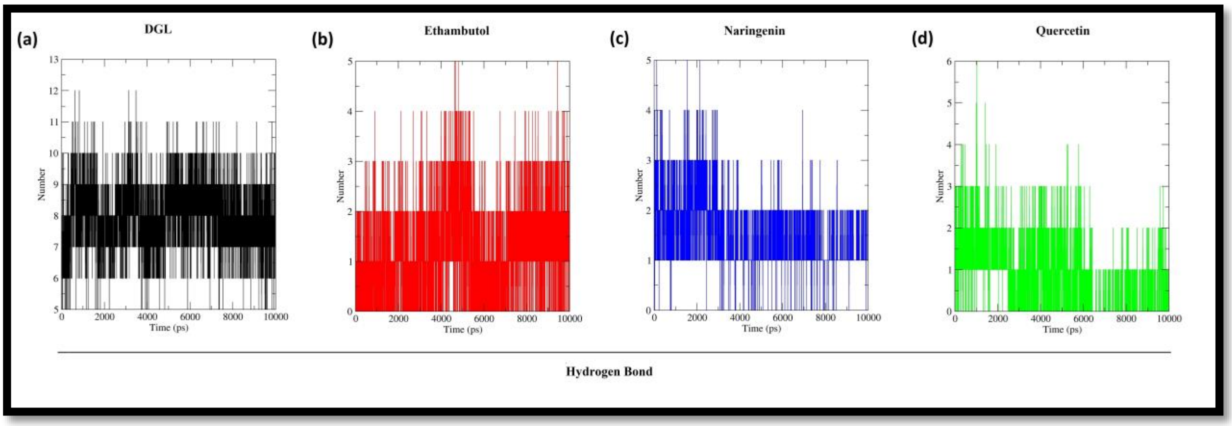

Fig. S3: Total number of intermolecular H-bonds between the ligand and enzyme complex versus time at 300K

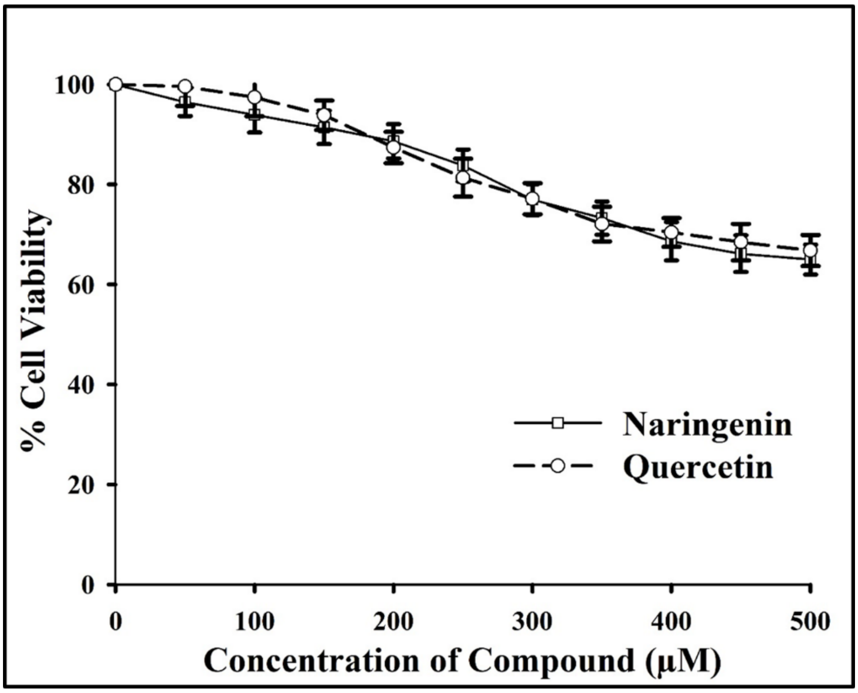

Fig. S4: Cytotoxicity graph showing toxic profile of naringenin and quercetin using THP-I monocytic macrophage cell lines. Reduction of THP-1 cells in response to increasing concentrations of flavonoids. Dose response curves of THP-1 cells was constructed after 48h treatment with naringenin and quercetin. The cells viability was assayed by the colorimetric MTT based assay. The X-axis is in logarithmic scale. Values were expressed as Mean ± SEM of three independent replicates.
